# Association of blood cell‐based inflammatory markers with gut microbiota and cancer incidence in the Rotterdam study

**DOI:** 10.1002/cam4.6860

**Published:** 2024-02-17

**Authors:** Shiva Najjary, Johan M. Kros, Bruno H. Stricker, Rikje Ruiter, Yu Shuai, Robert Kraaij, Kristel Van Steen, Peter van der Spek, Casper H. J. Van Eijck, M. Arfan Ikram, Shahzad Ahmad

**Affiliations:** ^1^ Department of Pathology and Clinical Bioinformatics, The Tumor Immuno‐Pathology Laboratory Erasmus University Medical Center Rotterdam the Netherlands; ^2^ Department of Epidemiology Erasmus University Medical Center Rotterdam the Netherlands; ^3^ Department of Internal Medicine Erasmus University Medical Center Rotterdam the Netherlands; ^4^ Department of Human Genetics, Laboratory for Systems Medicine Center for Human Genetics, KU Leuven Leuven Belgium; ^5^ Department of Surgery Erasmus University Medical Center Rotterdam the Netherlands

**Keywords:** cancer risk, gut microbiota, immune response, inflammatory markers, systemic inflammation index (SII)

## Abstract

The immune response–gut microbiota interaction is implicated in various human diseases, including cancer. Identifying the link between the gut microbiota and systemic inflammatory markers and their association with cancer will be important for our understanding of cancer etiology. The current study was performed on 8090 participants from the population‐based Rotterdam study. We found a significant association (false discovery rate [FDR] ≤0.05) between lymphocytes and three gut microbial taxa, namely the family Streptococcaceae, genus *Streptococcus*, and order *Lactobacillales*. In addition, we identified 95 gut microbial taxa that were associated with inflammatory markers (*p* < 0.05). Analyzing the cancer data, we observed a significant association between higher systemic immune‐inflammation index (SII) levels at baseline (hazard ratio (HR): 1.65 [95% confidence interval (CI); 1.10–2.46, *p* ≤ 0.05]) and a higher count of lymphocytes (HR: 1.38 [95% CI: 1.15–1.65, *p* ≤ 0.05]) and granulocytes (HR: 1.69 [95% CI: 1.40–2.03, *p* ≤ 0.05]) with increased risk of lung cancer after adjusting for age, sex, body mass index (BMI), and study cohort. This association was lost for SII and lymphocytes after additional adjustment for smoking (SII = HR:1.46 [95% CI: 0.96–2.22, *p* = 0.07] and lymphocytes = HR: 1.19 [95% CI: 0.97–1.46, *p* = 0.08]). In the stratified analysis, higher count of lymphocyte and granulocytes at baseline were associated with an increased risk of lung cancer in smokers after adjusting for age, sex, BMI, and study cohort (HR: 1.33 [95% CI: 1.09–1.62, *p* ≤0.05] and HR: 1.57 [95% CI: 1.28–1.92, *p* ≤0.05], respectively). Our study revealed a positive association between gut microbiota, higher SII levels, and higher lymphocyte and granulocyte counts, with an increased risk of developing lung cancer.

## Graphical abstract

To evaluate the association between gut microbiota and the immune response markers NLR, PLR, SII, and WBCs count with the subsequent risk of developing cancer in the Rotterdam study cohort (ERGO). The current study was performed on 8090 participants from the population‐based Rotterdam study. A significant association was found between lymphocytes and three gut microbial taxa, namely the family Streptococcaceae, genus Streptococcus, and order Lactobacillales. By analyzing the cancer data, a significant association was observed between higher systemic immune‐inflammation index (SII) levels at baseline and a higher count of lymphocytes and granulocytes with increased risk of lung cancer after adjusting for age, sex, BMI, and study cohort. However, this association was lost for SII and lymphocytes after additional adjustment for smoking. In the stratified analysis, higher count of lymphocyte and granulocytes at baseline were associated with an increased risk of lung cancer in smokers after adjusting for age, sex, BMI, and study cohort. Our study revealed a positive association between gut microbiota, higher SII levels, and higher lymphocyte and granulocyte counts, with an increased risk of developing lung cancer.

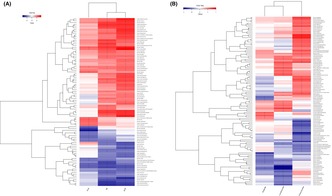

## 1 BACKGROUND

Gut microbiota influences the development and modulation of the innate and adaptive immune systems of the host by stimulating circulatory immune cells and cytokines. Under healthy conditions, gut microbes are symbiotic, contribute to the formation of stable homeostasis^1^ and are involved in the regulation of gut integrity and permeability.^2^ Interactions between the gut microbiota, intestinal epithelial cells, and mucosal immune system are believed to prevent the overgrowth of host flora by foreign pathogens.^3^, ^4^, ^5^ Several factors, such as dietary behavior, medication, autoimmune diseases, and infection, may influence the diversity of the gut microbiota,^6^, ^7^, ^8^ which may in turn influence their role in human physiology. Loss of balance between the gut microbiota, intestinal epithelial barrier, and immune system may lead to a pathological state known as gut microbial dysbiosis.^9^ Although dysbiosis is not considered to have a direct influence on carcinogenic pathways,^10^, ^11^, ^12^, ^13^ it can negatively affect the host immune system via various signaling pathways, including tumor‐infiltrating lymphocytes and their related cytokines, toll‐like receptors (TLRs), and innate immune cells.^14^, ^15^, ^16^, ^17^ Immune dysregulation and inflammation play a role in various diseases, including cancer.^10^ Inflammation may stimulate tumorigenesis and contribute to accumulated DNA damage, but may also indicate the presence of a tumor that has not yet been apparent.^13^ In both cases, products of the inflammatory response may serve as potential prognostic and diagnostic biomarkers for cancer risk.^13^, ^18^, ^19^, ^20^ At this point, our understanding of the role of microbiota in the development of diseases via modulation of the immune system in humans remains limited owing to the difficulty of direct experimentation.

The measurement of granulocytes, platelets, and lymphocytes is an important marker of the innate immune response.^20^, ^21^ Combining these measurements into ratios, including the platelet‐to‐lymphocyte ratio (PLR), granulocyte‐to‐lymphocyte ratio (NLR), and systemic immune‐inflammation index (SII), is believed to reflect the balance between innate and adaptive immunity.^22^, ^23^ Previous studies have reported the role of the gut microbiota in immune responses and their association with the development of various cancers.^20^, ^24^ However, which of the human gut microbial taxa is associated with immune response remains to be determined.

The aim of our study was to evaluate the association between gut microbiota and the immune response markers NLR, PLR, SII, and WBCs count in the population‐based Rotterdam study cohort (ERGO). Furthermore, we investigated the association between the levels of immune response markers and WBC count with the subsequent risk of developing cancer in the Rotterdam study cohort.

## 2 METHODS

### 2.1 Study population

The current study was embedded within the Rotterdam study (RS), a prospective population‐based cohort study in Rotterdam, Netherlands. The RS started in 1990 with 7983 individuals aged ≥55 years. The participants resided in a well‐defined district of Ommoord in the Rotterdam area. The initial cohort (RS‐I) was extended with a second cohort (RS‐II) in 2000, composed of 3011 individuals aged ≥55 years, and a third cohort (RS‐III) in 2006, consisting of 3932 individuals aged ≥45 years. The fourth cohort (RS‐IV) was established in February 2016.^25^, ^26^ The RS included 14,926 individuals aged ≥45 years. The participants underwent interviews at home and were examined at study entry and at follow‐up visits every 3–5 years.^25^ Health status and anthropometric and clinical variables were assessed in a standardized manner by trained research nurses and physicians in a specially built research facility in the center of the district.^27^ WBC count measurements and laboratory tests for the inflammatory markers NLR, PLR, and SII have been introduced since 2002, including 8711 participants corresponding to the following assessment rounds in the RS (baseline for this study): fourth round of RS‐I (RS‐I‐4), second round of RS‐II (RS‐II‐2 follow‐up), and first round of RS‐III (RS‐III‐1 follow‐up). To study gut microbiota, stool samples were collected from RS‐III‐2 participants.

Data from 8090 participants were included in the analysis. Participants with missing WBC counts or those who were diagnosed with cancer before the initial blood collection at baseline were excluded (Figures S1 and S2).

### 2.2 Assessment of gut microbiota

Details of the collection and sequencing of the RS samples have been described previously.^28^ The barcodes were separated from the reads and both ends of the sequences were attached together. These barcodes were used to multiplex the reads for each sample, which allowed one error per 12‐nucleotide half of the 24‐nucleotide long barcode using an in‐house developed script. The reads were then cleaned of heterologous primer sequences and spacers using a tag cleaner version 0.16.^29^ The trimmed reads were imported into the DADA2 R package (version 1.18.0).^30^ Samples without reads after the previous steps were removed, and the remaining reads were used as inputs for the DADA2 filter step. In this step of filtering, reads with an expected error rate >2 in both forward/reverse reads, and reads with at least one or more ambiguous bases in them (“N”) were removed. Additionally, reads were truncated if a low‐quality baseline was observed (*Q*‐score <2). The reads were clustered and denoised based on similarity, starting with the most abundant reads. Other reads that had a similarity of 10% and a frequency *p*‐value below the default threshold were included and aligned to this cluster. The algorithm was repeated with the remaining reads for the next abundant cluster. The nature of the algorithm requires multiples of the same read; therefore, singletons are automatically removed. Each denoised pair of forward and reverse reads was merged if there was 100% overlap between them. Reads that were truncated in the DADA2 filter step were removed because of a lack of overlap. Next, chimeric sequences were identified and removed by removing the Bimera Denovo using the consensus method in DADA2. The clustered clean reads, called amplicon sequence variant (ASV), were then used as an input for the RDP naive Bayesian classifier, which was trained on the SILVA Project version 138.1.^31^, ^32^ Taxonomy for Kingdom through genus was assigned for the bootstrap confidence >50, meaning that a random portion of the read could be assigned to that taxa at least 50% of the time. Species assignment was reported only if the ASV could be 100% matched to one species and could not be mapped to other species. The ASV table, taxonomy table, and metadata were then merged into a phyloseq.^33^ Both abundance and prevalence filters were applied to the data to remove spurious ASVs and possible false positives. ASVs had to have at least 0.05% of the total reads to remain in the dataset and be present in at least 1% of the samples, otherwise they were removed. At this stage of the pipeline, samples were also excluded based on other criteria, including being possible sample swaps, ≥8 days, time in mail, known duplicates, or QC statistics (for those samples with less than 4.5 K reads or samples missing more than 50% of reads in the last steps of DADA2_QC; i.e., those with a large number of reads but were actually sparsely distributed in ASV) were removed from the data. Also, samples with 4.5K–6K reads that lost more than 20% of reads in the last steps of the DADA2‐QC were excluded. Alpha and beta diversity statistics were calculated based on this phyloseq. Additionally, a phylogenetic tree was constructed based on the central sequences of each ASV using the phangorn package, and the results were added to the phylogenetic tree.^34^ Finally, ASV IDs were recoded from their central sequences to numerical IDs ordered by ASV abundance in the population.

### 2.3 Assessment of white blood cell‐based inflammatory markers

Blood samples were collected during the visit to the research center, and a maximum of three visits were made during the follow‐up. Blood samples were collected from April 10, 2002 (RS‐I‐4) to January 1, 2006 (RS‐II‐2) and from March 7, 2006 until January 20, 2009 (RS‐III‐1). Full blood count measurements were performed directly after blood sampling using a COULTER® Ac·T diff2™ Hematology Analyzer (Beckman Coulter, San Diego, CA, USA). Laboratory measurements included absolute granulocyte, platelet, and lymphocyte counts of 10^9^ per liter. The NLR and PLR were calculated as the granulocyte‐to‐lymphocyte and platelet‐to‐lymphocyte count ratios, respectively. The SII was defined as the platelet count multiplied by the NLR.^35^

### 2.4 Assessment of cancer in the Rotterdam Study

Incident diagnosis of cancer was the outcome of interest. Cancer cases were obtained from the medical records of general practitioners (including discharge letters), Dutch hospital data, and the histological and cytopathological record registries in the region. Each case was coded independently by two physicians and classified according to the International Classification of Diseases, 10th revision (ICD‐10). Information on cancer from the Rotterdam study was available until January 2, 2018. In case of discrepancies, a consensus was reached through consultation with an internal medicine physician. The date of diagnosis was based on the date of pathology confirmation.

### 2.5 Statistical analysis

#### 2.5.1 Association of gut microbiota with immune markers and white blood cells

Before the association analysis of gut microbiota with immune markers and WBCs, we performed a central log transformation to the gut microbial taxa ASV variables. We tested 1759 microbial taxa from seven taxonomic ranks: kingdom, phylum, class, order, family, genus, and species. The data underwent natural log transformation to reduce the skewness of the distribution of WBC counts (lymphocytes, granulocytes, and platelets; Figure S3) and their derived ratios (NLR, PLR, and SII; Figure S4). The relationship between microbial taxa, inflammatory markers, and WBCs count was investigated using a linear regression model adjusted for confounding factors (age, sex, BMI, alcohol consumption, smoking, and antibiotic use) and technical covariates (time in mail, DNA sequencing batch, and DNA isolation batch). Multiple testing was performed based on a FDR ≤0.05 for each biomarker and WBC.^36^

#### 2.5.2 Association of immune markers and white blood cells with risk of cancer development

Before the analysis, the log WBCs count was standardized (mean = 0, SD = 1) to make their estimates comparable. The association between the levels of immune markers at baseline and WBC count and the risk of solid cancers, including colorectal, pancreatic, breast, and lung cancers, and melanoma during follow‐up was evaluated using the Cox proportional hazard model adjusted for age, sex, BMI, and study cohort. Additionally, we examined the association after adjusting for smoking in the same model. For each individual, cancer follow‐up was specified by years from baseline until the date of diagnosis, death, or the end of the study (January 2, 2018). To evaluate the effect of smoking on the relationship between cancer and immune marker WBCs, we performed a stratified analysis based on smoking information, adjusting for age, sex, BMI, and study cohort. The results are presented as HR with 95% CI. Results with a *p* ≤0.05 were considered statistically significant. All analyses were performed using R (version 4.2) and IBM SPSS Statistics, version 28.0.

## 3 RESULTS

### 3.1 Demographics

The mean age of the participants at baseline was 65.25 years (standard deviation [SD] 10.41 years). The group comprised of 57.5% women (*N* = 4649). The mean values of the immune markers were: NLR 0.56 (SD, 0.39); PLR 4.78 (SD, 0.35), and SII 6.12 (SD, 0.46). The mean values of WBCs included: lymphocytes 0.78 (SD 0.30); granulocytes 1.34 (SD 0.32), and platelets 5.56 (SD 0.25). The characteristics of each cohort are presented in Table 1.

**TABLE 1:** Baseline characteristics of participants included in the current study.

Characteristic	Total	All	RS‐I	RS‐II	RS‐III
Number, *n* (%)	Individuals	8090 (100)	2696 (33.3)	2017 (24.9)	3377 (41.7)
Gender, *n* (%)	Male	3441 (42.5)	1078 (40)	872 (43.2)	1491 (44.2)
	Female	4649 (57.5)	1618 (60)	1145 (56.8)	1886 (55.8)
Age (years)	Mean (SD)	65.25 (10.41)	74.88 (6.16)	67.29 (7.01)	56.33 (6.62)
Smoking status, *n* (%)	Never smoking	2555 (32)	837 (31.8)	633 (32)	1085 (32.2)
	Former smoker	4022 (50.4)	1486 (56.4)	1038 (52.5)	1498 (44.5)
	Current smoker	1404 (17.6)	313 (11.9)	305 (15.4)	786 (23.3)
BMI (kg/m^2^)	Mean (SD)	27.66 (4.34)	27.41 (4.11)	27.41 (4.11)	27.75 (4.63)
White blood cells, Mean (SD)	Lymphocytes	0.78 (0.30)	0.66 (0.31)	0.80 (0.29)	0.85 (0.28)
	Granulocytes	1.34 (0.32)	1.35 (0.30)	1.32 (0.32)	1.34 (0.34)
	Platelets	5.56 (0.25)	5.51 (0.26)	5.53 (0.24)	5.62 (0.23)
Inflammatory immune markers, mean (SD)	NLR	0.56 (0.39)	0.68 (0.39)	0.51 (0.38)	0.48 (0.37)
	PLR	4.78 (0.35)	4.84 (0.38)	4.73 (0.34)	4.76 (0.32)
	Index (SII)	6.12 (0.46)	6.19 (0.48)	6.05 (0.46)	6.11 (0.44)
History of cancer, *n* (%)	No cancer	7129 (88.1)	2241 (83.1)	1721 (85.3)	3167 (93.8)
	Colorectal	166 (2.1)	88 (3.3)	43 (2.1)	35 (1.0)
	Breast	127 (1.6)	49 (1.8)	41 (2.0)	37 (1.1)
	Lung	121 (1.5)	64 (2.4)	35 (1.7)	22 (0.7)
	Pancrease	28 (0.3)	11 (0.4)	8 (0.4)	9 (0.3)
	Melanoma	22 (0.3)	11 (0.4)	6 (0.3)	5 (0.1)
	Other cancers	497 (6.1)	232 (8.6)	163 (8.1)	102 (3.0)

Abbreviations: SD, standard deviation; NLR, neutrophil‐to‐lymphocyte ratio; PLR, platelet‐lymphocyte ratio; index SII, systemic immune‐inflammation index.

### 3.2 Gut microbiota with inflammatory immune markers

**FIGURE 1:**
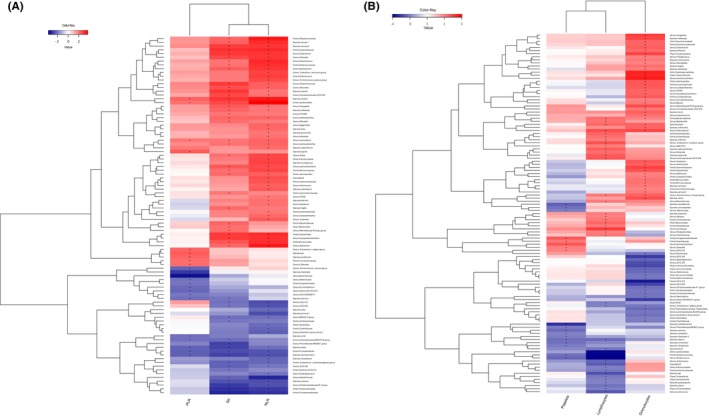
(A) Association analysis between gut microbiota and inflammatory immune markers NLR, PLR, and SII assessed by the linear regression model. (B) Association analysis between gut microbiota and white blood cells assessed by the linear regression model. Heatmaps indicate the association results after adjustment for confounding factors (age, sex, BMI, alcohol, smoking, and antibiotic use) and technical covariates (time in mail, DNA sequencing batch, and DNA isolation batch).

**FIGURE 2:**
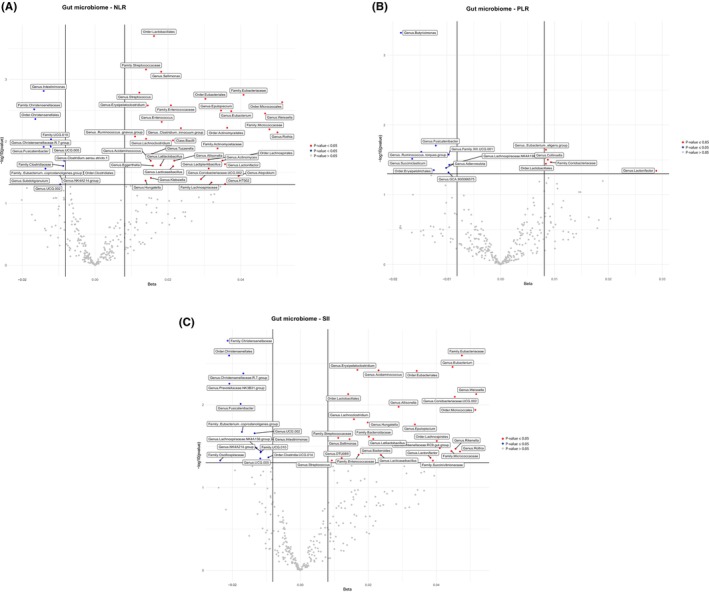
Association analysis between gut microbiota and inflammatory immune marker NLR, PLR, SII. Volcano plots of the beta coefficient on the *x*‐axis and −log10 (*p*‐value) on the *y*‐axis shows the association between NLR (A), PLR (C), SII (D), and microbial composition. Red and Blue dots represent microbial genera and families that are associated with the related immune marker (*p* < 0.05). The red dots show positive and blue dots indicate inverse association. The gray dots show no association (*p* > 0.05).

A list of all microbial taxa assessed for their association with inflammatory immune markers (NLR, PLR, and SII) after adjusting for cofactors is shown in Figures 1A and 2, and Table S1. A total of 95 microbial families and genera were associated with the inflammatory immune markers NLR, PLR, and SII (Figures 1A and 2, and Table S2). In total, 61 taxa had a positive association and 34 taxa had an inverse association with the immune markers NLR, PLR, and SII (Figures 1A and 2, and Table S2). Most of these microbial families and genera were from the phylum Firmicutes, whereas the others were from the phyla *Actinobacteriota* and *Bacteroidota* (Table S2). Thirty‐three microbial taxa were positively associated with a single immune marker: 20 taxa with NLR, six with PLR, and seven with SII. Increased levels of all three immune markers were associated with increased abundance of the order *Lactobacillales*, genus *Lactonifactor* and species, whereas decreased levels of all three biomarkers were associated with decreased abundance of the genus *Fusicatenibacter*, species *shahii*, and species *saccharivorans* (Table S3). Twenty‐five microbial taxa were positively associated with these two immune markers (Table S4).

### 3.3 Gut microbiota with white blood cells

**FIGURE 3:**
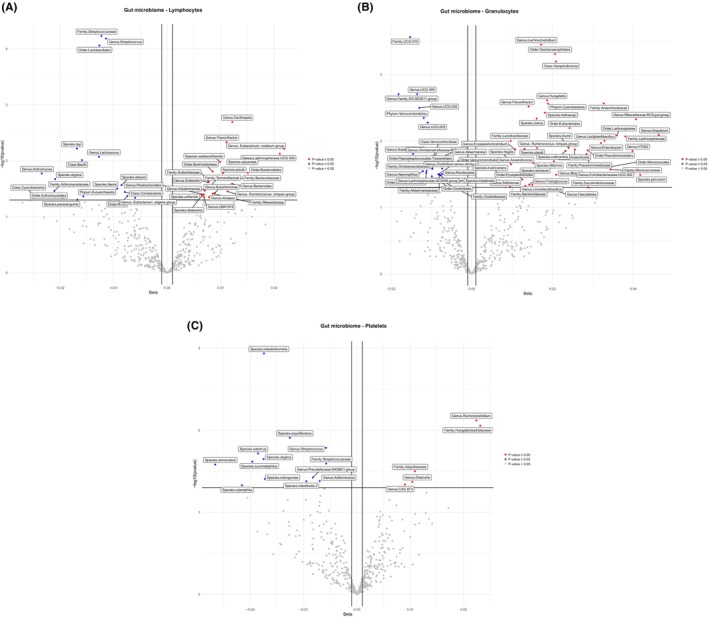
Association analysis between gut microbiota and white blood cells (lymphocytes, granulocytes, and platelets). Volcano plots of the beta coefficient on the *x*‐axis and − log10 (*p*‐value) on the *y*‐axis indicates the association between lymphocytes (A), granulocytes (B), platelets (C), and microbial composition. Red and Blue dots show microbial genera and families that are associated with the related WBC (*p* < 0.05). The red dots indicate positive and blue dots shows inverse association (*p* < 0.05). The gray dots show no association (*p* > 0.05).

**FIGURE 4:**
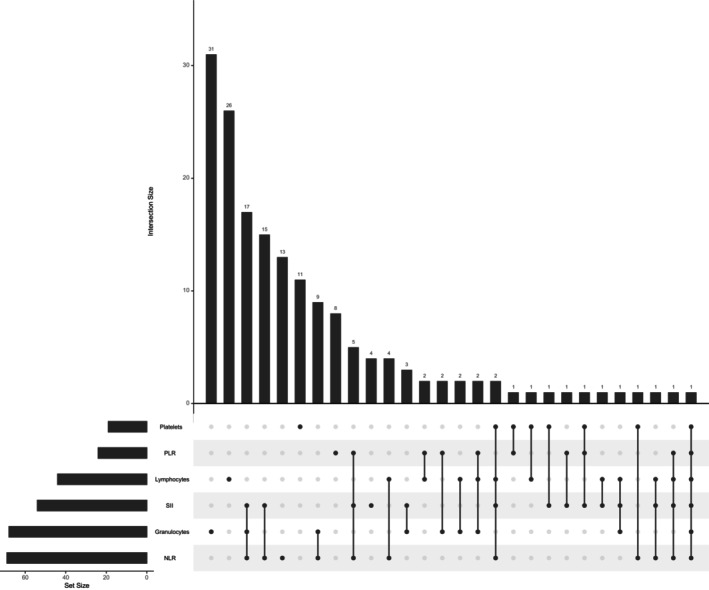
UpSet plot indicating the overlap of gut microbial taxa between immune markers of NLR, PLR, SII, and white blood cells (lymphocytes, granulocytes, and platelets).

A complete list of microbial taxa evaluated for their association with WBCs (lymphocytes, granulocytes, and platelets) after adjusting for cofactors is shown in (Figures 1B and 3, and Table S5). After removing duplicates, we identified 120 microbial families and genera associated with lymphocytes, granulocytes, and platelets (Figures 1B and 3, and Table S6). Importantly, we observed three microbial taxa, including the family Streptococcaceae, the order *Lactobacillales*, and the genus *Streptococcus* which were inversely associated with lymphocytes (false discovery rate [FDR] ≤ 0.05). All three microbial families and genera belong to the phylum Firmicutes (Figures 1B and 3, and Table S6). Overall, 70 taxa had a positive association and 50 taxa had an inverse association with lymphocytes, granulocytes, and platelets. 66 microbial taxa were positively associated with a single WBC count: 41 taxa with granulocytes, 20 with lymphocytes, and 5 with platelets. Four microbial taxa were positively associated with two WBCs counts (Table S7). The overlap between WBCs count and immune markers for gut microbial taxa is shown in Figure 4.

### 3.4 Risk of cancer development

A total, 961 individuals (11.9%) developed cancer during follow‐up, namely cancers of colorectal (*n* = 166, 17.3%), breast (*n* = 127, 13.2%), lung (*n* = 121, 12.6%), pancreatic (*n* = 28, 2.9%), other cancers (*n* = 497, 51.7%), and melanoma (*n* = 22, 2.3%). Higher baseline SII levels appeared to be specifically associated with an increased risk of lung cancer after adjusting for age, sex, body mass index (BMI), and study cohort (hazard ratios [HR]: 1.65 [95% confidence interval, CI 1.10–2.46], *p* ≤ 0.05; Table 2). This association disappeared after additional adjustment for smoking (HR: 1.46 [95% CI: 0.96–2.22], *p* = 0.07; Table 3). No association was observed between the NLR and PLR and the incidence of any of the cancers included in this analysis (Tables 2 and 3). In addition, high lymphocyte and granulocyte counts were a significantly associated with an increased risk of lung cancer after adjusting for age, sex, BMI, and study cohort with lymphocytes (HR: 1.38 [95% CI: 1.15–1.65, *p* ≤0.05]) and granulocytes (HR: 1.69 [95%CI: 1.40–2.03, *p* ≤0.05]; Table 4). This association disappeared for lymphocytes after additional adjustment for smoking (HR: 1.19 [95% CI: 0.97–1.46], *p*= 0.08; Table S8).

**TABLE 2:** Multivariate Cox regression analysis for the association between baseline PLR, NLR, and SII level with the development of cancer in Model 1.

Cancer type	*N*	NLR		PLR		SII	
		Hazard ratio (95% CI)^a^	*p*‐value	Hazard ratio (95% CI)^a^	*p*‐value	Hazard ratio (95% CI)^a^	*p*‐value
Colorectal	163	1.12 (0.74–1.69)	0.58	1.12 (0.71–1.77)	0.60	1.28 (0.92–1.79)	0.13
Breast	124	0.98 (0.61–1.58)	0.95	1.24 (0.72–2.13)	0.42	1.05 (0.70–1.55)	0.80
Lung	110	1.59 (0.96–2.62)	0.06	0.64 (0.37–1.09)	0.10	1.65 (1.10–2.46)	0.01
Pancreas	25	1.28 (0.45–3.66)	0.63	0.80 (0.25–2.57)	0.71	1.03 (0.43–2.43)	0.93
Melanoma	21	1.93 (0.61–6.07)	0.25	2.07 (0.58–7.40)	0.25	2.12 (0.84–5.31)	0.10

^a^ Adjusted for age (years), sex, BMI, and RS cohort.

**TABLE 3:** Multivariate Cox regression analysis for the association between baseline PLR, NLR, and SII level with the development of cancer in Model 2.

Cancer type	*N*	NLR		PLR		SII	
		Hazard ratio (95% CI)^a^	*p*‐value	Hazard ratio (95% CI)^a^	*p*‐value	Hazard ratio (95% CI)^a^	*p*‐value
Colorectal	156	1.13 (0.74–1.73)	0.56	1.05 (0.66–1.67)	0.83	1.26 (0.90–1.78)	0.17
Breast	124	0.97 (0.60–1.57)	0.91	1.30 (0.75–2.24)	0.34	1.04 (0.70–1.54)	0.83
Lung	108	1.36 (0.81–2.29)	0.23	0.91 (0.52–1.60)	0.75	1.46 (0.96–2.22)	0.07
Pancreas	24	1.17 (0.40–3.43)	0.77	0.88 (0.26–2.93)	0.84	0.97 (0.40–2.34)	0.95

^a^ Adjusted for age (years), sex, BMI, RS cohort, and smoking.

**TABLE 4:** Multivariate Cox regression analysis for the association between white blood cell count with the development of cancer based on Model 1.

Cancer type	*N*	Lymphocytes		Granulocytes		Platelets	
		Hazard ratio (95% CI)^a^	*p*‐value	Hazard ratio (95% CI)^a^	*p*‐value	Hazard ratio (95% CI)^a^	*p*‐value
Colorectal	163	1.07 (0.91–1.25)	0.40	1.12 (0.95–1.32)	0.15	1.15 (0.98–1.36)	0.08
Breast	124	0.95 (0.78–1.15)	0.64	0.95 (0.79–1.14)	0.63	1.05 (0.87–1.28)	0.56
Lung	110	1.38 (1.15–1.65)	3 × 10^−4^	1.69 (1.40–2.03)	3.2 × 10^−8^	1.21 (0.99–1.48)	0.057
Pancreas	25	0.98 (0.64–1.49)	0.93	1.10 (0.74–1.65)	0.62	0.88 (0.58–1.32)	0.54
Melanoma	21	0.94 (0.59–1.48)	0.79	1.29 (0.82–2.04)	0.25	1.37 (0.86–2.18)	0.18

^a^ Adjusted for age (years), sex, BMI, RS‐cohort.

### 3.5 Effects of smoking

The results of the association between immune markers (NLR, PLR, and SII) and WBCs count with smoking status and BMI are shown in Table 5 and Table S9, respectively. As BMI and smoking were found to be major drivers of the association between immune markers and WBCs with cancer development, we assessed the individual role of BMI and smoking in the blood levels of immune markers (NLR, PLR, and SII) and WBCs by linear regression adjusted for age and sex. All three immune markers and WBCs were significantly associated with smoking after adjusting for age and sex, namely NLR (0.08, 95% CI: 0.056–0.106, *p =* 1.99 × 10^−10^), PLR (−0.13, 95% CI: [(−0.156) to (−0.111)], *p* < 2 × 10^−16^), SII (0.11, 95% CI: 0.083–0.144, *p* = 2.93 × 10^−13^), lymphocytes (0.54, 95% CI: 0.480–0.605, *p* < 2 × 10^−16^), granulocytes (0.75, 95% CI: 0.693–0.819, *p* < 2 × 10^−16^), and platelets (0.12, 95% CI: 0.062–0.187, *p* = 8.17 × 10^−5^). The levels of PLR and all three WBCs correlated with those of BMI after adjusting for age and sex, with PLR (−0.008, 95%CI [(−0.010) to (−0.006)], *p* < 2 × 10^−16^), lymphocytes (0.02, 95% CI: 0.018–0.028, *p* <2 × 10^−16^), granulocytes (0.02, 95% CI: 0.022–0.032, *p* < 2 × 10^−16^), and platelets (−0.005, 95% CI: [(−0.010) to (−0.0004)], *p* = 0.03).

**TABLE 5:** Linear regression analysis for the association between baseline PLR, NLR, and SII level with smoking status and BMI.

Model	Variable	NLR		PLR		SII	
		Hazard ratio (95% CI)^a^	*p*‐value	Hazard ratio (95% CI)^a^	*p*‐value	Hazard ratio (95% CI)^a^	*p*‐value
Smoking	Former smoker	0.01 (−0.002–0.036)	0.082	−0.003 (−0.020–0.014)	0.718	0.02 (−0.0003–0.0467)	0.053
	Current smoker	0.08 (0.056–0.106)	1.99 × 10^−10^	−0.13 (−0.156 to −0.111)	<2 × 10^−16^	0.11 (0.083–0.144)	2.93 × 10^−13^
BMI	BMI	0.001 (−9.260–0.003)	0.062	−0.008 (−0.010 to −0.006)	<2 × 10^−16^	0.0005 (−0.001–0.002)	0.662

^a^ Adjusted for age (years) and sex.

In the stratified analysis, 260 (9.8%) individuals among nonsmokers (*n* = 2664) developed cancer during follow‐up, viz., colorectal cancers (*n* = 56, 21.5%), breast cancer (*n* = 50, 19.2%), lung cancer (*n* = 13, 5%), pancreatic cancer (*n* = 11, 4.2%), melanoma (*n* = 11, 4.2%), and other cancers (*n* = 119, 45.8%). The baseline characteristics of the respective cohorts of non‐smokers are shown in Table S10. Among smokers (*n* = 5426), 701 individuals (12.9%) developed cancer during follow‐up viz., cancers of colon and rectum (*n* = 110, 15.7%), breast (*n* = 77, 11%), lung (*n* = 108, 15.4%), pancreas (*n* = 17, 2.4%), melanoma (*n* = 11, 1.6%), and other cancers (*n* = 378, 53.9%). The characteristics of each cohort of smokers are shown in Table S13. We observed a significant association between higher counts of granulocytes and platelets with increased risk of colorectal cancer (HR: 1.41 [95% CI: 1.05–1.89, *p* ≤ 0.05] and HR: 1.59 [95% CI: 1.17–2.17, *p* ≤ 0.05], respectively) and a higher count of granulocytes with increased risk of lung cancer (HR: 1.82 [95% CI: 1.00–3.32, *p* ≤ 0.05]) in nonsmoker group after adjusting for age, sex, BMI, and study cohort (Table S11). However, we found no effect of NLR, PLR, or SII on the risk of lung cancer, colorectal cancer, pancreatic cancer, breast cancer, and melanoma in the nonsmoker group after adjusting for age, sex, BMI, and study cohort (Table S12). We observed a significant association between lymphocytes and granulocytes an increased risk of lung cancer in smokers after adjusting for age, sex, BMI, and study cohort (HR: 1.33 [95% CI: 1.09–1.62, *p* = 3.53 × 10^−3^], and HR: 1.57 [95% CI: 1.28–1.92, *p* = 9.61 × 10^−6^], respectively; Table 6). Moreover, higher baseline SII levels were associated with increased risk of lung cancer in smokers after adjusting for age, sex, BMI, and study cohort (HR: 1.61 [95% CI: 1.05–2.47, *p* ≤ 0.05]; Table S14).

**TABLE 6:** Multivariate Cox regression analysis for the association between lymphocytes, granulocytes, and platelets counts with the development of cancer in smokers.

*Cancer type*	*N*	Lymphocytes		Granulocytes		Platelets	
		Hazard ratio (95% CI)^a^	*p*‐value	Hazard ratio (95% CI)^a^	*p*‐value	Hazard ratio (95% CI)^a^	*p*‐value
Colorectal	109	0.96 (0.79–1.18)	0.75	1.00 (0.82–1.23)	0.92	0.99 (0.81–1.20)	0.93
Breast	75	0.96 (0.75–1.23)	0.75	0.91 (0.72–1.15)	0.44	1.08 (0.83–1.39)	0.54
Lung	98	1.33 (1.09–1.62)	3.53 × 10^−3^	1.57 (1.28–1.92)	9.61 × 10^−6^	1.22 (0.99–1.50)	0.057
Pancreas	17	0.85 (0.51–1.42)	0.54	0.83 (0.52–1.35)	0.47	0.83 (0.53–1.31)	0.44
Melanoma	10	0.70 (0.35–1.37)	0.30	1.15 (0.60–2.22)	0.66	1.20 (0.60–2.38)	0.59

^a^ Adjusted for age (years), sex, BMI, RS‐cohort.

## 4 DISCUSSION

Gut microbiota dysbiosis can negatively affect the host immune system causing immune dysregulation and inflammation. Various factors, including dietary behavior, autoimmune disorders, medication, and infection can impact the gut microbiota diversity, influencing their role in human physiology.^6^, ^37^ The immune dysregulation and inflammation may influence the development and progression of tumorigenesis and cancer.^10^, ^38^ Importantly, the inflammatory response products could serve as potential biomarkers for cancer risk and diagnosis.^39^, ^40^ Our current understanding of how microbiota modulates the immune system in humans and its role in disease development remains limited, primarily due to the challenges of direct experimentation. Therefore investigating the association between gut microbiota and immune response in developing diseases such as cancer is important. In the present study, we evaluated the association of WBCs and inflammatory immune markers (NLR, PLR, and SII) with the gut microbiota and cancer development. Our study provides evidence of an association between 120 microbial taxa and WBCs counts (*p* < 0.05). Specifically, we found a significant association between three gut microorganisms from the phylum Firmicutes and lymphocytes (FDR ≤0.05), namely, the family Streptococcaceae, the genus *Streptococcus*, and the order *Lactobacillales*. In addition, we identified an association between the 95 microbial taxa and NLR, PLR, and SII (*p* < 0.05). However, these associations did not pass the significance threshold FDR of 0.05. Among the identified associations, 61 gut microorganisms from the phyla *Firmicutes*, *Bacteroidota*, *Actinobacteriota*, and *Proteobacteria* were positively associated with the inflammatory immune markers.

The gut microbial taxa associated with high WBCs count and increased inflammatory response in our study were cancer‐related microorganisms reported in earlier studies,^41^, ^42^ indicating the role of the gut microbiome in cancer development via the inflammatory response.^43^ Alterations in the abundance of these microbes influence the initiation and progression of gastrointestinal carcinogenesis^44^ but may also affect the immune system, which can lead to the development of extraintestinal malignancies.^45^, ^46^ Several studies have reported a relationship between the high abundance of these microbial taxa (phylum *Firmicutes*, *Bacteroidota*, *Actinobacteriota*, and *Proteobacteria*) and cancer risk.^47^, ^48^, ^49^, ^50^ Firmicutes are one of the largest phyla of gram‐positive bacteria living in the human intestine^51^ and are associated with obesity and several pathological conditions.^52^, ^53^ Among the taxa in this phylum, the genus *Lachnoclostridium*,^54^ order *Lactobacillales*,^55^ family Streptococcaceae,^55^ genus *Streptococcus*,^56^ and genus *Eubacterium* ^54^ have been reported to be more abundant in patients with lung carcinoma than in healthy individuals. In addition, elevated abundance of the genus *Eubacterium* ^54^ and genus *Streptococcus* ^56^ have also been indicated to be related to lung cancer. We found a positive association between WBC count and the genus *Lachnoclostridium*, family Streptococcaceae, genus *Streptococcus* and genus *Eubacterium*, with WBCs count (*p* < 0.05). Interestingly, we observed an inverse association between the order *Lactobacillales*, family Streptococcaceae, and genus Streptococcus with WBCs count (*p* < 0.05), which passed the significance threshold of FDR ≤0.05 for lymphocytes. Additionally, we found a positive association between these taxa and blood biomarkers NLR and SII (*p* < 0.05). Moreover, we found a positive association between the order *Actinomycetales* and WBCs count and the immune marker NLR. The order *Actinomycetales*, belonging to the phylum *Actinomycetota* and class *Actinomycetia* are anaerobic, prokaryotic filamentous, Gram‐positive bacteria found in soil as well as in humans and animals. These bacteria are important for maintaining homeostasis in the human gut.^57^, ^58^ The enriched abundance of *Actinomycetales* has been associated with lung and colorectal cancers.^46^, ^55^ These data and our results provide an in‐depth understanding of the role of the gut microbiome in the inflammatory immune response and its impact on the development of various cancers.

In addition to the role of gut dysbiosis in regulating the immune response, our data revealed an association between inflammatory immune markers and WBCs, and the risk of developing cancer. We found that individuals from the general population with higher baseline SII levels and higher lymphocyte and granulocyte counts were more likely to develop cancer during the follow‐up period. A significant association was observed between high levels of SII and high lymphocyte and granulocyte counts with the risk of lung cancer after adjusting for covariates, including age, sex, BMI, and study‐specific cohort. However, the significance of this association was lost upon adjustment for smoking status. The significant association between SII and WBCs and current smoking in our study shows that the association between SII and WBCs and lung cancer is mainly driven by smoking. Several studies have reported an association between high levels of SII and high WBCs count and an increased risk of cancers, including lung cancer.^19^, ^59^, ^60^ In a UK biobank‐based study, not only a positive relationship between the SII but also between the NLR and PLR and the risk of developing cancer was demonstrated.^61^ Tian et al. reported a relationship between SII and PLR in colorectal tumors.^62^ Fest et al. revealed that higher SII levels at baseline were associated with a higher risk of lung, colorectal, bladder, and prostate cancers.^20^ In another UK biobank‐based study, elevated WBCs counts were found to be associated with an increased risk of lung cancer in women who had never smoked and men who smoked or had a history of smoking.^63^ Lee et al. indicated that high WBCs counts were associated with a higher incidence and mortality risk of colon cancer, with a positive linear trend in non‐smokers.^64^ Giannakeas et al. showed that high platelet counts are associated with colon, lung, and ovarian cancers.^65^ We also found a significant association between granulocytes and platelets and an increased risk of colorectal cancer in nonsmokers. Taken together, the results of the current study suggest that WBC count may be a useful cancer screening tool alone or in combination with other screening methods. The significant association between a high WBC count and colorectal cancer in non‐smokers suggests that the increased risk of colorectal cancer is independent of the effect of smoking on WBCs. Furthermore, given that WBC count and SII are immune‐inflammatory markers that reflect systemic inflammation, alterations in microbial taxa may be associated with immune dysregulation and inflammation, which could affect the development of various tumors.

We could not evaluate the levels of SII and WBC count at different stages of cancer because the data were not available. Despite this limitation, our study is one of the largest population‐based studies showing an association between gut microbiota, inflammatory immune responses, and cancer development. The most obvious finding to emerge from this study was that high levels of SII and high lymphocyte and granulocyte counts were associated with the development of lung cancer over time.

In conclusion, our results revealed a positive association between the pathogenic gut microbiota and inflammatory immune responses that could promote cancer development. We identified that alterations in baseline SII and WBC count could be independent risk indicators for early detection of the disease. Further studies are needed to validate this association and to evaluate its potential for clinical management.

## AUTHOR CONTRIBUTIONS

**Shiva Najjary:** Data curation (lead); formal analysis (lead); funding acquisition (lead); investigation (lead); methodology (lead); software (lead); validation (lead); visualization (lead); writing – original draft (lead); writing – review and editing (lead). **Johan M. Kros:** Conceptualization (equal); project administration (equal); supervision (equal); writing – review and editing (equal). **Bruno H. Stricker:** Data curation (equal); resources (equal); writing – review and editing (equal). **Rikje Ruiter:** Data curation (equal); resources (equal); writing – review and editing (equal). **Yu Shuai:** Data curation (equal); resources (equal); writing – review and editing (equal). **Robert Kraaij:** Data curation (equal); resources (equal); writing – review and editing (equal). **Kristel Van Steen:** Investigation (equal); validation (equal); writing – review and editing (equal). **Peter van der Spek:** Investigation (equal); validation (equal); writing – review and editing (equal). **Casper H. J. van Eijck:** Investigation (equal); validation (equal); writing – review and editing (equal). **M. Arfan Ikram:** Investigation (equal); validation (equal); writing – review and editing (equal). **Shahzad Ahmad:** Conceptualization (equal); project administration (equal); supervision (equal); writing – review and editing (equal).

## FUNDING INFORMATION

This study was financially supported by the European Union's Horizon 2020 Research and Innovation Program under the Marie Sklodowska‐Curie grant agreement no. 860895 TranSYS.

## CONFLICT OF INTEREST STATEMENT

The authors declare no potential conflicts of interest.

## ETHICS APPROVAL AND CONSENT TO PARTICIPATE

The RS was approved by the Medical Ethics Committee of the Erasmus MC under registration number MEC 02.1015 and by the Dutch Ministry of Health, Welfare, and Sport. All participants included in this study provided written informed consent to participate and obtain their information from the treating physicians.

## Supporting information


Material S1.

Material S2.

Material S3.

## Data Availability

Data were obtained by submitting a request to the Rotterdam Study Management Team (secretariat.epi@erasmusmc.nl), which has a protocol to approve the data request. Data cannot be made publicly available due to restrictions based on privacy policies and the informed consent of participants.
