# Supplementary material for: Association of blood cell‐based inflammatory markers with gut microbiota and cancer incidence in the Rotterdam study
Source: Cancer Med. 2024 Feb 17;13(3):e6860. doi: 10.1002/cam4.6860 (PMC10904974; doi:10.1002/cam4.6860)
Supplement: Supplementary file 2 — Material S2. [file CAM4-13-e6860-s003.docx]

**Supplementary Table S8.** Multivariate Cox regression analysis for the association between white blood cell count with the development of cancer in Model 2.

| **Cancer type** | **N** | **Lymphocytes** | | **Granulocytes** | | **Platelets** | |
| --- | --- | --- | --- | --- | --- | --- | --- |
|  |  | **Hazard Ratio (95% CI)^1^** | ***P*-value** | **Hazard Ratio (95% CI)^1^** | ***P*-value** | **Hazard Ratio (95% CI)^1^** | ***P*-value** |
| **Colorectal** | 156 | 1.08 (0.91-1.28) | 0.32 | 1.15 (0.97-1.37) | 0.09 | 1.13 (0.95-1.34) | 0.14 |
| **Breast** | 124 | 0.93 (0.77-1.13) | 0.51 | 0.93 (0.77-1.27) | 0.47 | 1.05 (0.87-1.28) | 0.57 |
| **Lung** | 108 | 1.19 (0.97-1.46) | 0.08 | 1.36 (1.11-1.66) | 2.68×10^-3^ | 1.17 (0.95-1.42) | 0.11 |
| **Pancreas** | 24 | 0.94 (0.60-1.46) | 0.79 | 1.02 (0.67-1.57) | 0.90 | 0.88 (0.58-1.33) | 0.55 |
| **Melanoma** | 20 | 1.01 (0.63-1.61) | 0.95 | 1.40 (0.87-2.26) | 0.16 | 1.47 (0.91-2.38) | 0.11 |

^1^Adjusted for age (year), sex, BMI, smoking, RS-cohort.

**Supplementary Table S9.** Linear regression analysis for the association between baseline white blood cell count with smoking status and BMI.

| **Model** | **Variables** | **Lymphocytes** | | **Granulocytes** | | **Platelets** | |
| --- | --- | --- | --- | --- | --- | --- | --- |
|  |  | **Hazard Ratio (95% CI)^1^** | ***P*-value** | **Hazard Ratio (95% CI)^1^** | ***P*-value** | **Hazard Ratio (95% CI)^1^** | ***P*-value** |
| **Smoking** | **Former smoker** | 0.02 (-0.018-0.078) | 0.22 | 0.08 (0.031-0.128) | 1.23 × 10^-3^ | 0.02 (-0.024-0.072) | 0.32 |
|  | **Current smoker** | 0.54 (0.480-0.605) | <2×10^-16^ | 0.75 (0.693-0.819) | <2×10^-16^ | 0.12 (0.062-0.187) | 8.17×10^-5^ |
| **BMI** | **BMI** | 0.02 (0.018-0.028) | <2×10^-16^ | 0.02 (0.022-0.032) | <2×10^-16^ | -0.005 (-0.010 - -0.0004) | 0.03 |

^1^Adjusted for age (year) and sex.

**Supplementary Table S10.** Baseline characteristics of non-smoker participants in the current study.

| **Characteristic** | **Total** | **All** | **RS-I** | **RS-II** | **RS-III** |
| --- | --- | --- | --- | --- | --- |
| Number, n (%) | Individuals | 2664 (100) | 897 (33.7) | 674 (25.3) | 1093 (41) |
| Gender, n (%) | Male | 747 (28) | 140 (15.6) | 189 (28) | 329 (20.9) |
|  | Female | 1917 (72) | 757 (84.4) | 485 (72) | 1242 (79.1) |
| Age (years) | Mean (SD) | 65.62 (11) | 75.64 (6.65) | 67.72 (7.88) | 56.10 (6.68) |
| BMI (in kg/m^2^) | Mean (SD) | 27.66 (4.35) | 27.75 (4.21) | 27.40 (4.02) | 27.73 (4.65) |
| White blood cells, Mean (SD) | Lymphocytes | 0.74 (0.29) | 0.67 (0.30) | 0.76 (0.29) | 0.80 (0.26) |
|  | Granulocytes | 1.27 (0.32) | 1.30 (0.31) | 1.24 (0.32) | 1.26 (0.31) |
|  | Platelets | 5.57 (0.23) | 5.54 (0.24) | 5.55 (0.23) | 5.61 (0.22) |
| Inflammatory immune markers, Mean (SD) | NLR | 0.52 (0.39) | 0.64 (0.40) | 0.49 (0.39) | 0.57 (0.41) |
|  | PLR | 4.82 (0.33) | 4.87 (0.35) | 4.79 (0.33) | 4.83 (0.35) |
|  | Index (SII) | 6.10 (0.46) | 6.18 (0.48) | 6.05 (0.46) | 6.1 (0.48) |
| History of cancer, n (%) | No cancer | 2404 (90.2) | 776 (86.5) | 587 (87.1) | 1363 (86.8) |
|  | Colorectal | 56 (2.1) | 26 (2.9) | 20 (3) | 46 (2.9) |
|  | Breast | 50 (1.9) | 22 (2.5) | 16 (2.4) | 38 (2.4) |
|  | Lung | 13 (0.5) | 9 (1.0) | 4 (0.6) | 13 (0.8) |
|  | Pancrease | 11 (0.4) | 7 (0.8) | 3 (0.4) | 10 (0.6) |
|  | Melanoma | 11 (0.4) | 7 (0.8) | 2 (0.3) | 9 (0.6) |
|  | Other cancers | 119 (4.5) | 50 (5.6) | 42 (6.2) | 92 (5.9) |

*Note.* SD: standard deviation ; NLR: Neutrophil-Lymphocyte Ratio ; PLR: platelet-lymphocyte ratio ; Index (SII): systemic immune-inflammation index.

**Supplementary Table S11.** Multivariate Cox regression analysis for the association between white blood cell count with the development of cancer in non-smokers.

| **Cancer type** | **N** | **Lymphocytes** | | **Granulocytes** | | **Platelets** | |
| --- | --- | --- | --- | --- | --- | --- | --- |
|  |  | **Hazard Ratio (95% CI)^1^** | ***P*-value** | **Hazard Ratio (95% CI)^1^** | ***P*-value** | **Hazard Ratio (95% CI)^1^** | ***P*-value** |
| **Colorectal** | 47 | 1.31 (0.98-1.75) | 0.06 | 1.41 (1.05-1.89) | 0.02 | 1.59 (1.17-2.17) | 2.91 × 10^-3^ |
| **Breast** | 49 | 0.90 (0.66-1.22) | 0.51 | 0.97 (0.72-1.29) | 0.83 | 1.03 (0.76-1.41) | 0.82 |
| **Lung** | 10 | 1.55 (0.84-2.88) | 0.15 | 1.82 (1.00-3.32) | 0.04 | 1.20 (0.61-2.35) | 0.59 |
| **Pancreas** | 7 | 1.23 (0.56-2.68) | 0.60 | 1.82 (0.82-4.02) | 0.13 | 0.97 (0.41-2.26) | 0.95 |
| **Melanoma** | 10 | 1.34 (0.74-2.44) | 0.32 | 1.57 (0.83-3.00) | 0.16 | 1.81 (0.95-3.44) | 0.06 |

^1^Adjusted for age (year), sex, BMI, RS-cohort.

**Supplementary Table S12.** Multivariate Cox regression analysis for the association between baseline PLR, NLR, and SII level with the development of cancer in non-smokers.

| **Cancer type** | **N** | **NLR** | | **PLR** | | **Index (SII)** | |
| --- | --- | --- | --- | --- | --- | --- | --- |
|  |  | **Hazard Ratio (95% CI)^1^** | ***P*-value** | **Hazard Ratio (95% CI)^1^** | ***P*-value** | **Hazard Ratio (95% CI)^1^** | ***P*-value** |
| **Colorectal** | **47** | 1.17 (0.56 - 2.44) | 0.67 | 1.18 (0.50-2.78) | 0.70 | 1.73 (0.94-3.16) | 0.07 |
| **Breast** | **49** | 1.12 (0.54-2.32) | 0.76 | 1.37 (0.57-3.28) | 0.46 | 1.11 (0.61-2.04) | 0.71 |
| **Lung** | **10** | 1.48 (0.29-7.39) | 0.62 | 0.46 (0.07-2.84) | 0.40 | 1.55 (0.41-5.82) | 0.51 |
| **Pancreas** | **7** | 2.09 (0.30-14.60) | 0.45 | 0.57 (0.06-5.26) | 0.62 | 1.65 (0.31-8.70) | 0.55 |
| **Melanoma** | **10** | 1.29 (0.26-6.23) | 0.74 | 1.34 (0.21-8.34) | 0.75 | 2.08 (0.57-7.65) | 0.26 |

^1^Adjusted for age (year), sex, BMI, RS-cohort.

**Supplementary Table S13.** Baseline characteristics of participants with smoking history in the current study.

| **Characteristic** | **Total** | **All** | **RS-I** | **RS-II** | **RS-III** |
| --- | --- | --- | --- | --- | --- |
| Number, n (%) | Individuals | 5426 (100) | 1799 (33.2) | 1343 (24.8) | 2284 (42.1) |
| Gender, n (%) | Male | 2694 (49.6) | 938 (52.1) | 683 (50.9) | 1073 (47) |
|  | Female | 2732 (50.4) | 861 (47.9) | 660 (49.1) | 1211 (53) |
| Age, years | Mean (SD) | 65.06 (10.10) | 74.51 (5.87) | 67.06 (6.51) | 56.44 (6.59) |
| Smoking status, n (%) | Former smoker | 4022 (74.1) | 1486 (82.6) | 1038 (77.3) | 1498 (65.6) |
|  | Current smoker | 1404 (25.9) | 313 (17.4) | 305 (22.7) | 786 (34.4) |
| BMI, in kg/m^2^ | Mean (SD) | 27.66 (4.30) | 27.32 (4.04) | 28.01 (4.12) | 27.71 (4.56) |
| White blood cells, Mean (SD) | Lymphocytes | 0.79 (0.31) | 0.67 (0.31) | 0.82 (0.28) | 0.88 (0.28) |
|  | Granulocytes | 1.37 (0.32) | 1.37 (0.29) | 1.35 (0.32) | 1.38 (0.34) |
|  | Platelets | 5.56 (0.25) | 5.49 (0.27) | 5.52 (0.24) | 5.62 (0.23) |
| Inflammatory immune markers, Mean (SD) | NLR | 0.57 (0.39) | 0.70 (0.38) | 0.53 (0.37) | 0.50 (0.37) |
|  | PLR | 4.76 (0.35) | 4.82 (0.39) | 4.70 (0.34) | 4.74 (0.33) |
|  | Index (SII) | 6.13 (0.46) | 6.20 (0.48) | 6.06 (0.46) | 6.13 (0.44) |
| History of cancer, n (%) | No cancer | 4725 (87.1) | 1465 (81.4) | 1134 (84.4) | 2126 (93.1) |
|  | Colorectal | 110 (2) | 62 (3.4) | 23 (1.7) | 25 (1.1) |
|  | Breast | 77 (1.4) | 27 (1.5) | 25 (1.9) | 25 (1.1) |
|  | Lung | 108 (2) | 55 (3.1) | 31 (2.3) | 22 (1.0) |
|  | Pancrease | 17 (0.3) | 4 (0.2) | 5 (0.4) | 8 (0.4) |
|  | Melanoma | 11 (0.2) | 4 (0.2) | 4 (0.3) | 3 (1.1) |
|  | Other cancers | 378 (7) | 182 (10.1) | 121 (9) | 75 (3.3) |

*Note.* SD: standard deviation ; NLR: Neutrophil-Lymphocyte Ratio ; PLR: platelet-lymphocyte ratio ; Index (SII): systemic immune-inflammation index.

**Supplementary Table S14.** Multivariate Cox regression analysis for the association between baseline PLR, NLR, and SII level with the development of cancer in smokers.

| **Cancer type** | **N** | **NLR** | | **PLR** | | **Index (SII)** | |
| --- | --- | --- | --- | --- | --- | --- | --- |
|  |  | **Hazard Ratio (95% CI)^1^** | ***P*-value** | **Hazard Ratio (95% CI)^1^** | ***P*-value** | **Hazard Ratio (95% CI)^1^** | ***P*-value** |
| **Colorectal** | **109** | 1.09 (0.64 - 1.83) | 0.74 | 1.06 (0.61-1.83) | 0.83 | 1.08 (0.71-1.64) | 0.70 |
| **Breast** | **75** | 0.86 (0.46-1.62) | 0.65 | 1.27 (0.63-2.54) | 0.49 | 0.98 (0.58-1.65) | 0.96 |
| **Lung** | **98** | 1.50 (0.87-2.58) | 0.13 | 0.73 (0.41-1.30) | 0.29 | 1.61 (1.05-2.47) | 0.02 |
| **Pancreas** | **17** | 0.88 (0.24-3.16) | 0.85 | 0.98 (0.24-3.99) | 0.97 | 0.75 (0.27-2.12) | 0.59 |
| **Melanoma** | **10** | 2.71 (0.52-14.07) | 0.23 | 3.22 (0.51-20.33) | 0.21 | 2.29 (0.61-8.54) | 0.21 |

^1^Adjusted for age (year), sex, BMI, and RS-cohort.
