# Supplementary material for: Association of blood cell‐based inflammatory markers with gut microbiota and cancer incidence in the Rotterdam study
Source: Cancer Med. 2024 Feb 17;13(3):e6860. doi: 10.1002/cam4.6860 (PMC10904974; doi:10.1002/cam4.6860)
Supplement: Supplementary file 3 — Material S3. [file CAM4-13-e6860-s002.pdf]

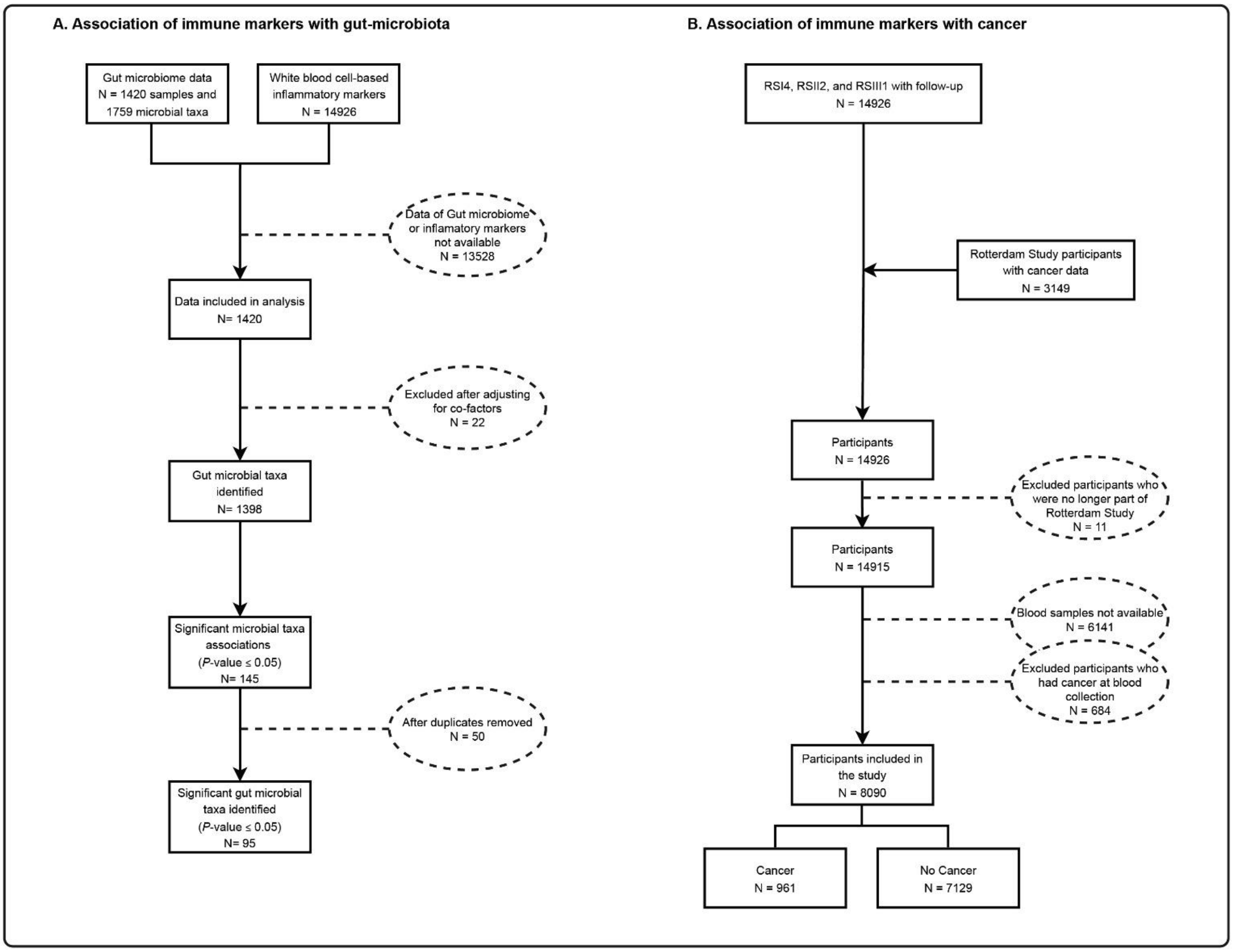

Figure S1. Flowchart of the association analysis for immune markers, gut-microbiota and cancer. (A) Association of immune markers with gut-microbiota. (B) Association of immune markers with cancer.

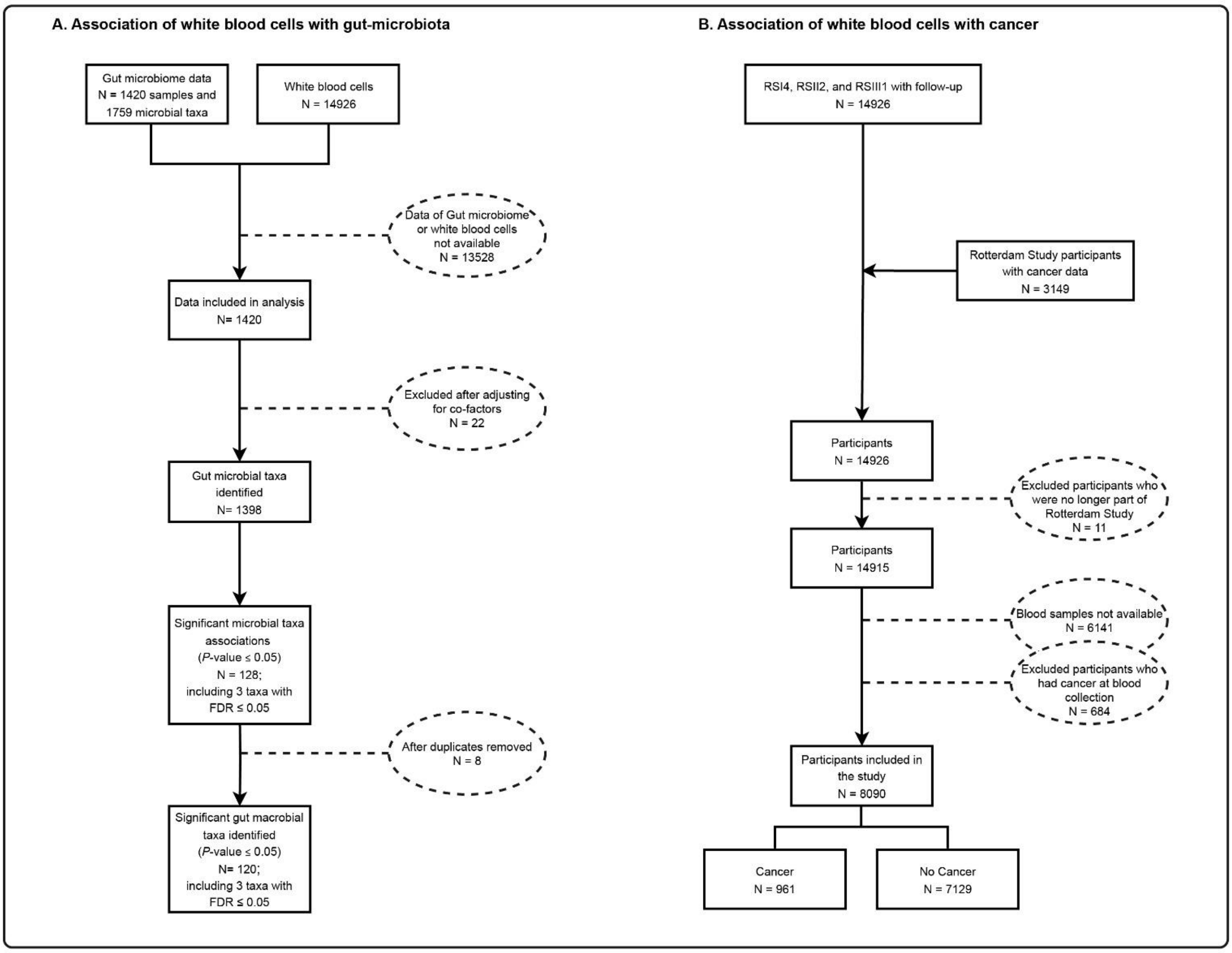

Figure S2. Flowchart of the association analysis for white blood cells, gut-microbiota and cancer. (A) Association of white blood cells with gut-microbiota. (B) Association of white blood cells with cancer.

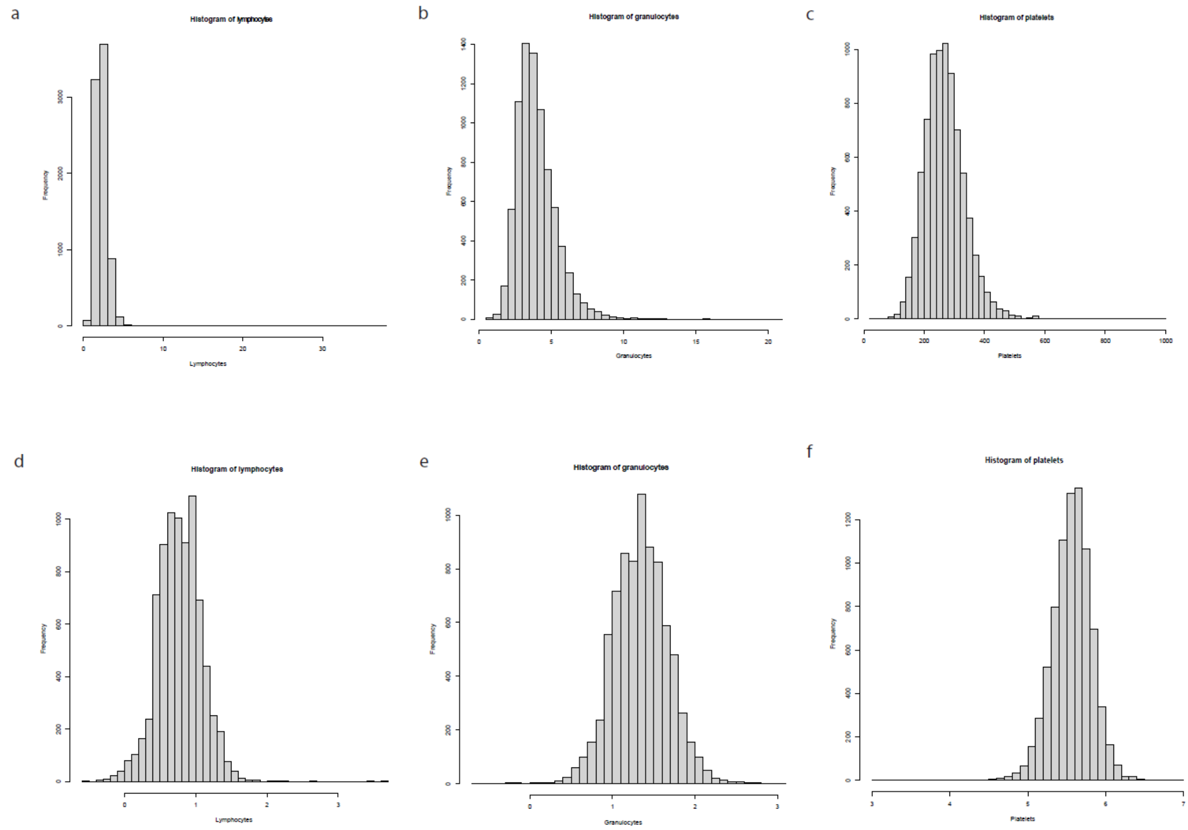

Figure S3. Histograms of the white blood cells before and after natural log transformation. (a) Lymphocytes, (b) Granulocytes, and (c) Platelets count before log transformation. (d) Lymphocytes, (e) Granulocytes, and (f) Platelets count after log transformation.

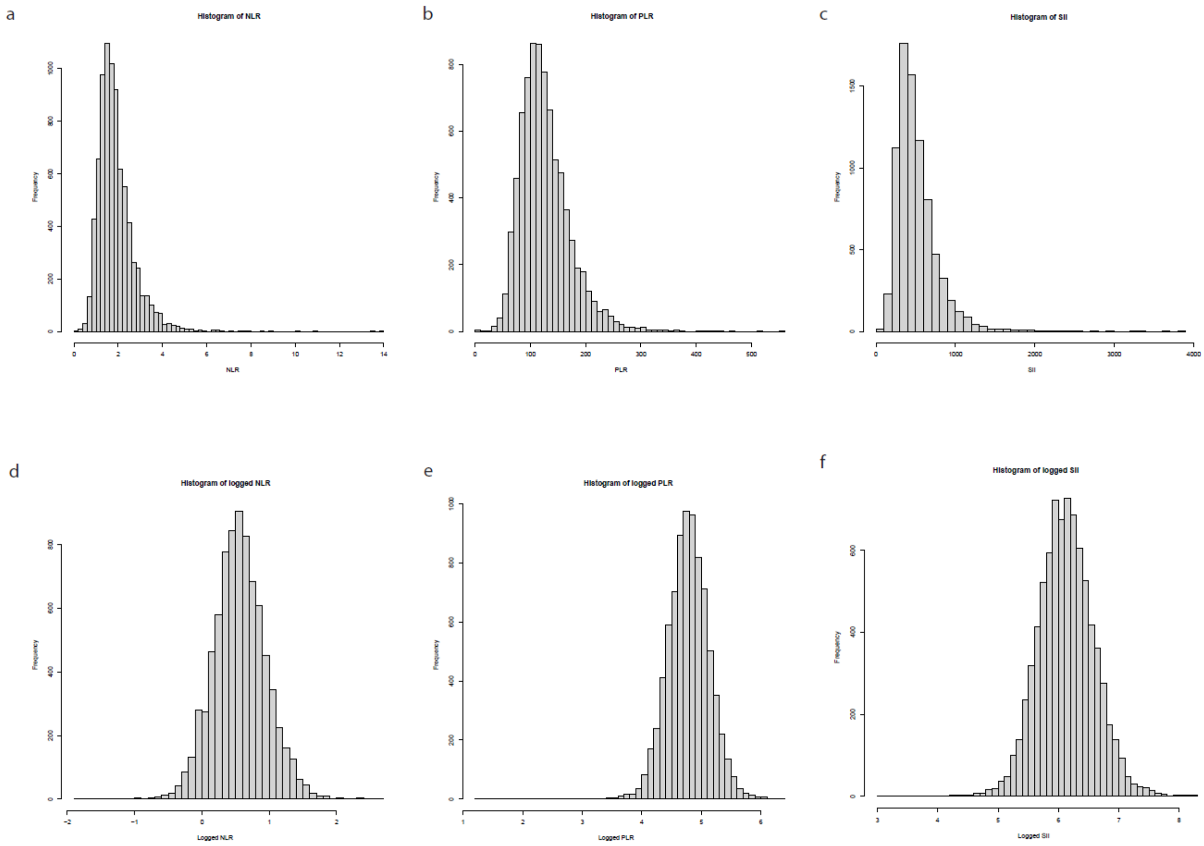

Figure S4. Histograms of the immune markers before and after natural log transformation. (a) NLR, (b) PLR, and (c) SII before log transformation. (d) NLR, (e) PLR, (f) SII after log transformation.
